# Context-dependent coordination of TOR and SnRK1 signaling under carbon and nitrogen perturbations

**DOI:** 10.3389/fpls.2026.1862048

**Published:** 2026-06-11

**Authors:** Hui Liu, Hai Shi, Jorg Schwender, John Shanklin, Zhiyang Zhai

**Affiliations:** Department of Biology, Brookhaven National Laboratory, Upton, NY, United States

**Keywords:** *Arabidopsis thaliana*, carbon and nitrogen availability, nutrient signaling, SnRK1, target of rapamycin

## Abstract

Target of rapamycin (TOR) and sucrose non-fermenting 1–related protein kinase 1 (SnRK1) are conserved regulators of plant growth and metabolism and are often portrayed as functionally antagonistic under nutrient limitation. However, how this relationship operates across different nutrient contexts remains poorly defined. Here, we generated an Arabidopsis dual-reporter line that enables simultaneous monitoring of TOR and SnRK1 activities and profiled their dynamics under carbon and nitrogen perturbations. We found that TOR and SnRK1 activities overall exhibit a negative relationship during the transition from carbon starvation to carbon abundance; however, their temporal dynamics during that transition do not support a strictly inverse correlation. Under dark conditions, TOR activity is gradually repressed, while SnRK1 is initially repressed in the early hours and subsequently activated during extended darkness. During nitrogen starvation, TOR activity is progressively repressed, whereas SnRK1 is activated during early hours and then becomes repressed. *In vitro*, recombinant SnRK1α1 directly inhibits the activity of immunoprecipitated TOR (IP-TOR), whereas IP-TOR does not directly affect SnRK1α1 activity. Together, these results support a nutrient-dependent model in which TOR and SnRK1 are coordinated primarily by cellular metabolic status.

## 1 Introduction

Plants, as sessile organisms, continuously experience fluctuations in nutrient availability and environmental conditions that necessitate dynamic metabolic adjustments. Two evolutionarily conserved protein kinase systems, target of rapamycin (TOR) and sucrose non-fermenting-1 related protein kinase 1 (SnRK1), act as central regulators in these adjustments. TOR promotes biosynthetic activity by stimulating ribosome biogenesis, protein translation, and meristem proliferation, while concurrently suppressing autophagy. In contrast, SnRK1 is activated under conditions of nutrient/energy limitation, where it enhances catabolic processes and restrains growth-related programs (Baena-González and Hanson, 2017; Rodriguez et al., 2019; Peixoto and Baena-González, 2022; Li and Zhao, 2024; Liu et al., 2025b). Because of these overall opposing roles, TOR and SnRK1 are frequently described as functioning in a “yin–yang” relationship. Supporting evidence includes: (1) *In vitro* phosphorylation of Regulatory-Associated Protein of TOR (RAPTOR) by the SnRK1 catalytic subunit SnRK1α1 (Nukarinen et al., 2016); (2) *In vivo* interaction between SnRK1 and TOR, with SnRK1 acting as a negative regulator of TOR activity (Belda-Palazon et al., 2020); (3) Transcriptomic studies showing antagonistic regulation of overlapping gene sets (Baena-González et al., 2007; Xiong et al., 2013; Dong et al., 2015), together with phosphoproteomic studies linking altered SnRK1 activity to changes in the phosphorylation of canonical TOR targets, such as S6K1 (ribosomal protein S6 kinase 1) and RPS6 (ribosomal protein S6) (Cho et al., 2016); (4) Opposing roles in autophagy regulation, with TOR acting as a suppressor of autophagy (Liu and Bassham, 2010) and SnRK1 as an activator (Chen et al., 2017); (5) Activity of TOR and SnRK1 demonstrating negative relationship in Arabidopsis PSB-D cell culture overexpressing S6K1-3×HA grown under nitrogen starvation conditions (Persyn et al., 2024); (6) FCS-like Zinc Finger protein FLZ8 acting as a TOR feedback node that promotes SnRK1-mediated inhibition of TOR under favorable conditions (Jamsheer et al., 2024).

However, accumulating evidence also suggests that the interplay between TOR and SnRK1 cannot be simplified to an antagonism. Genome-wide expression analyses reveal only partial overlap between TOR- and SnRK1- regulated targets (Wu et al., 2019), and TOR activity has been associated with improved tolerance to environmental stresses, including cold and drought (Bakshi et al., 2017; Dong et al., 2019). Moreover, crosstalk between the two pathways is also shaped by metabolic signals, such as trehalose-6-phosphate (T6P) (Morales-Herrera et al., 2023), and by multiple hormonal pathways, including abscisic acid (Wang et al., 2018; Belda-Palazon et al., 2020; Belda-Palazón et al., 2022). In addition, both TOR and SnRK1 were shown to potentiate SPEECHLESS (SPCH), a master regulator of stomatal development (Han et al., 2022), suggesting in the specific developmental context, TOR and SnRK1 function cooperatively.

In plants, phosphorylation of S6K1 and its downstream substrate RPS6 are well-established readouts of TOR *in vivo* activity. In yeast, Deroover et al (Deroover et al., 2016). designed a specific *in vivo* reporter for SNF1(sucrose non-fermenting 1) activity that incorporates a peptide containing a consensus recognition motif for SNF1/SnRK1/AMPK. This peptide was derived from rat acetyl-coA carboxylase 1 (ACC1), a well-characterized AMPK substrate (Dale et al., 1995). The phosphorylation state of the reporter can be assessed after protein extraction using immunoblotting with a validated phospho-specific antibody, allowing direct measurement of SNF1 activity *in vivo*. Adapted versions of this ACC1-based reporter were later applied in Arabidopsis to investigate how SnRK1 responds to nitrogen availability (Sanagi et al., 2021), light-dark cycles (Avidan et al., 2023), as well as to examine its role in other physiological processes (Muralidhara et al., 2021; Belda-Palazón et al., 2022; Henninger et al., 2022). In principle, combining S6K1/RPS6- and ACC1-based reporter under different promoters or targeting them to specific cellular compartments would allow monitoring of TOR and SnRK1 activities at the whole plant level, in distinct cell types, or within subcellular regions. However, a major limitation of these approaches is that they rely on endpoint measurement requiring tissue extraction. Alternatively, a Separation of Phase-based Activity Reporter of Kinase (SPARK) sensor has been successfully developed for SnRK1 (Persyn et al., 2024), enabling real-time monitoring SnRK1 activity in living tissues. Nevertheless, quantification of SPARK signals can be challenging, particularly when analyzing puncta that vary in number, size, and intensity. Moreover, to date, a SPARK sensor has not been reported for TOR.

Although substantial progress has been made in understanding the TOR-SnRK1 relationship, most studies have focused on the activity changes of either TOR or SnRK1 individually, and very few have monitored both pathways simultaneously in a relative short timeframe following experimental perturbations. To address this gap, we developed an Arabidopsis dual-reporter line that contains both a TOR substrate (S6K1-HA) and a SnRK1 substrate (rat ACC1 peptide) (Xiong and Sheen, 2012; Sanagi et al., 2021; Avidan et al., 2023) as activity readouts to simultaneously monitor TOR and SnRK1 activities. By applying conditions known to stimulate or repress each pathway, we tracked short-term activity dynamics. Our results show that TOR and SnRK1 exhibit a negative relationship primarily during the transition from carbon starvation to carbon abundance but are otherwise largely uncoupled *in vivo*. Furthermore, *in vitro* kinase assays using immunoprecipitated TOR and recombinant SnRK1α1 revealed that SnRK1α1 directly inhibits TOR activity, whereas TOR does not directly affect SnRK1α1 activity. Collectively, these results suggest that TOR influences SnRK1 primarily through indirect regulatory mechanisms rather than through a direct antagonistic interaction, and SnRK1-mediated inhibition of TOR represents only one component of TOR regulation, which is strongly influenced by cellular nutrient status.

## 2 Methods

### 2.1 Plant materials and growth conditions

*Arabidopsis thaliana* ecotype Columbia-0 (Col-0) was used as the wild type. The following transgenic or mutant lines were included in this study: an S6K1-HA overexpression line (ABRC stock CS73259) (Xiong and Sheen, 2012), a SnRK1 activity reporter line expressing two tandem rat ACC1 peptides fused to GFP and HA tags (GFP–2×ACC1–2×HA) (Sanagi et al., 2021). The S6K1-HA/ACC1 dual-reporter line (SA) was generated by crossing the S6K1-HA line with the ACC1 reporter line.

For seed germination under long-day conditions, seeds were surface sterilized and plated on half-strength Murashige and Skoog (½ MS) medium supplemented with 1% (w/v) sucrose and 0.4% (w/v) phytagel. Stratification was carried out for 3 d at 4 °C in darkness. Seedlings were grown in a controlled Percival growth chamber under a 16 h light/8 h dark cycle with temperatures of 24 °C during the light period and 22 °C at night, and a light intensity of 150 μmol m^-^² s^-^¹.

For the transition from carbon starvation to carbon abundance, three-day-old SA seedlings germinated on half-strength Murashige and Skoog (½ MS) medium containing 1% sucrose were transferred to ½ MS liquid medium and starved for 4 d in dark. Seedlings were then subsequently resupplied with 1% sucrose in the presence or absence of 1 μM TOR-specific inhibitor Torin2 and sampled at 0.5–4 h after sucrose resupply.

For dark conditions, seven-day-old S6K1-HA/ACC1 (SA) seedlings grown under a 12/12 h day/night photoperiod on the ½ MS were harvested at ZT8 (zeitgeber time, 8 h after the dawn), ZT16 (4 h into the dark), and ZT28 (16 h into the dark including 4 h of extended darkness).

For nitrogen deficiency, five-day-old S6K1-HA/ACC1 seedlings germinated on ½ MS solid medium (1% sucrose) containing 30 mM nitrogen were transferred to ½ MS liquid medium (1% sucrose) containing 30 mM or 0.3 mM or 0 mM nitrogen and harvested at 8 h, 1 d and 2 d.

### 2.2 Metabolite measurements

Metabolite extraction was adapted from (Mata et al., 2016) with minor modifications. Approximately 50 mg of fresh tissue was frozen in liquid nitrogen and homogenized using a Geno/Grinder. Metabolites were extracted in 500 μL of pre-chilled chloroform:methanol (3:7, v/v), followed by vortexing and incubation at −20 °C for 2 h with periodic mixing. Subsequently, 400 μL of ice-cold water was added, and samples were incubated at 4 °C for 15 min. Deoxy-glucose-6-phosphate (deoxy-G6P) was added as an internal standard. After centrifugation at 14,000 rpm for 10 min at 4 °C, the aqueous phase was collected, re-extracted, combined, and lyophilized.

Trehalose-6-phosphate (T6P) was quantified using a two-step derivatization procedure as described by Rende et al. (2019). Dried samples were dissolved in 100 μL of methoxylamine solution (20 mg mL^-^¹ in pyridine) and incubated at 60 °C for 30 min, followed by overnight incubation at room temperature. Subsequently, 30 μL of 1-methylimidazole and 60 μL of propionic anhydride were added, and the reaction proceeded at 37 °C for 30 min. Samples were dried under nitrogen gas and resuspended in 100 μL of 0.1% (v/v) formic acid prior to analysis.

Liquid chromatography–mass spectrometry (LC–MS) was performed using a Shimadzu Nexera X2 UHPLC system coupled to a Sciex QTRAP 4500 mass spectrometer operating in negative ion mode. Derivatized sugar phosphates were separated on an EVO C18 column (1.7 μm particle size, 100 Å pore size, 100 × 2.1 mm; Phenomenex). Sucrose measurements were conducted using a Luna NH_2_ column (100 × 2 mm, 3 μm; Phenomenex). The mobile phase consisted of solvent A (acetonitrile) and solvent B (20 mM ammonium acetate and 20 mM ammonium hydroxide in 95:5 water:acetonitrile, pH 9.45). The gradient program was as follows: 25% B at 0 min, 30% B at 8 min, 100% B from 22 to 32 min, and re-equilibration to 25% B from 33.5 to 44 min. The flow rate was maintained at 0.3 mL min^-^¹ and the column temperature at 40 °C. Quantification was performed using ^13^C_6_-glucose as an internal standard.

### 2.3 Production and purification of recombinant proteins

Coding sequences corresponding to SnRK1α1, GRIK1, HsEIF4EBP1, and constitutively active ROP2 (Q63L) were inserted into pET28a or pDEST-HisMBP vectors using In-Fusion cloning. Recombinant proteins carrying N-terminal His tags were expressed in *Escherichia coli* strain BL21 (DE3) and purified according to established procedures (Nallamsetty and Waugh, 2007).

### 2.4 Immunoblot analysis

Protein extraction and immunoblotting were conducted following previously described methods (Zhai et al., 2023). Approximately 20 mg of seedling tissue was ground in liquid nitrogen and resuspended in 80 μL of extraction buffer containing 8 M urea, 2% (w/v) SDS, 0.1 M DTT, 20% (v/v) glycerol, 0.1 M Tris-HCl (pH 6.8), and 0.004% (w/v) bromophenol blue. Samples were heated at 80 °C for 5 min, centrifuged at 17,000 × g, and 10 μL of the supernatant was separated by SDS–PAGE. Proteins were transferred onto PVDF membranes, blocked with 5% (w/v) nonfat milk, and incubated with primary antibodies including anti-TOR (1:3,000; Liu et al., 2025a), anti-phospho-acetyl-CoA carboxylase (Ser79; 1:1,000; Cell Signaling Technology, #3661) for detecting pACC-HA, anti-phospho-S6K1 (1:1,000; Agrisera, AS13 2664) for detecting pS6K1-HA, anti-HA (1:3,000; BioLegend, #901514), anti-phospho-RPS6 (1:5,000; Dobrenel et al., 2016), and anti-RPS6 (1:1,000; Cell Signaling Technology, #2317). Horseradish peroxidase–conjugated secondary antibodies (1:10,000; Agrisera, AS09 602) were detected using SuperSignal West Femto substrate (Thermo Fisher Scientific, #34095) on an Amersham ImageQuant 800 system.

### 2.5 Immunoprecipitation of native TOR

Immunoprecipitation of endogenous TOR was performed as described by (Fu et al., 2021). One gram of 7-d-old wild-type seedlings grown on ½ MS medium supplemented with 1% sucrose was frozen and ground in liquid nitrogen. Proteins were extracted in 5 mL of buffer containing 400 mM HEPES (pH 7.5), 2 mM EDTA, 10 mM sodium pyrophosphate, 10 mM glycerol phosphate, 0.3% (w/v) CHAPS, and a protease inhibitor cocktail (cOmplete Mini EDTA-free; Roche). Extracts were rotated at 4 °C for 15 min, clarified by centrifugation at 20,000 × g, and passed through Econo-Pac disposable columns. The supernatant was concentrated using 100 kDa Amicon Ultra centrifugal filters at 3,500 × g. Dynabeads Protein A were coupled to anti-TOR antibody and incubated with the extracts for 2 h at 4 °C. Beads were washed once with extraction buffer and twice with low-salt buffer (400 mM HEPES pH 7.5, 150 mM NaCl, 2 mM EDTA, 10 mM pyrophosphate, 10 mM glycerol phosphate, 0.3% CHAPS) before resuspension in kinase wash buffer (25 mM HEPES pH 7.5, 20 mM KCl).

### 2.6 TOR *in vitro* kinase assay

TOR kinase reactions were carried out in a final volume of 25 μL containing 25 mM HEPES (pH 7.5), 50 mM KCl, 10 mM MgCl_2_, 0.02 mM ATP, 1 μg of bead-bound immunoprecipitated TOR, and 1 μg of recombinant EIF4EBP1. Reactions were incubated at 30 °C for 15 min and stopped by addition of SDS sample buffer. TOR activity was assessed by immunoblot detection of phosphorylated EIF4EBP1 using a phospho-specific antibody recognizing Thr37/46 (Cell Signaling Technology, #2855). To evaluate the effect of SnRK1, recombinant SnRK1α1 (1 μg) or GRIK1-activated SnRK1α1 (1 μg SnRK1α1 plus 1 μg GRIK1) was added to the reaction. Negative controls lacked TOR or contained 1 μM Torin2, while positive controls included 1 μg of constitutively active ROP2.

### 2.7 SnRK1 *in vitro* kinase assay

SnRK1 kinase assays were conducted in 25 μL reactions containing 25 mM HEPES (pH 7.5), 50 mM KCl, 10 mM MgCl_2_, 0.02 mM ATP, 1 μg of SnRK1α1 or GRIK1-activated SnRK1α1, 0.2 mM SPS-derived peptide (RDHMPRIRSEMQIWSED), and 1 μCi of [γ-^32^P]ATP. Reactions were incubated at 30 °C for 5 min and terminated by spotting 10 μL onto 4 cm² phosphocellulose paper. Blotted papers were washed four times with phosphoric acid and once with acetone, dried, and placed into scintillation vials containing 2 mL of liquid scintillation cocktail (Filter-Count™, Revvity). Radioactivity was quantified using a Hidex 300 SL scintillation counter. To test the effect of TOR on SnRK1 activity, 1 μg of immunoprecipitated TOR was added to the assay. Control reactions included 1 μM Torin2.

### 2.8 Accession numbers

AT1G50030 (TOR), AT3G01090 (SnRK1α1), AT1G20090 (ROP2), AT3G45240 (GRIK1), AT3G08730 (S6K1), Q13541 (EIF4EBP1), AT4G31700 (RPS6).

## 3 Results

### 3.1 An Arabidopsis dual-reporter line for TOR and SnRK1 *in vivo* activity

**Figure 1:**
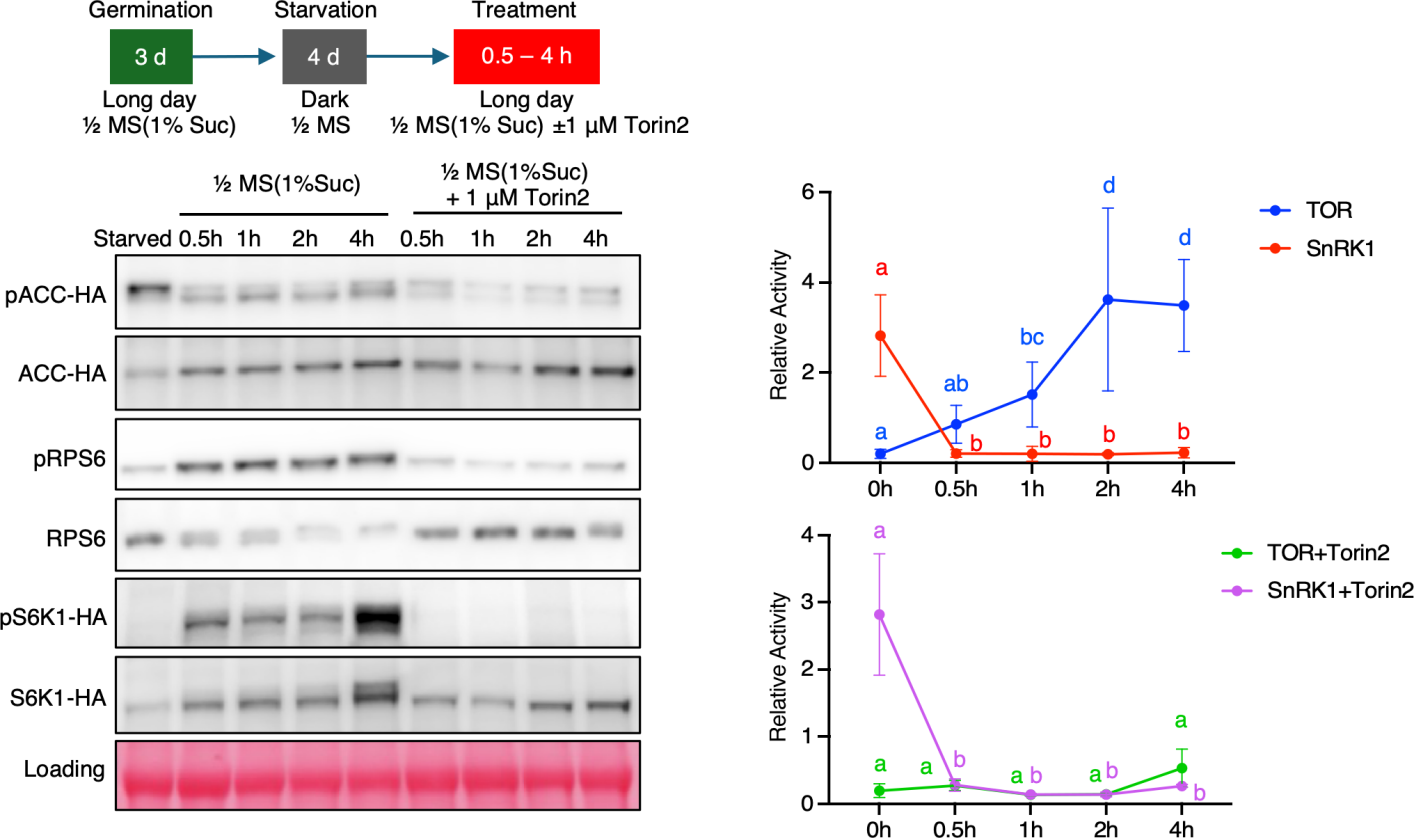
Negative relationship between TOR and SnRK1 activity during sucrose-mediated TOR activation. Starved S6K1-HA/ACC1 (SA) dual-reporter plants exhibit rapid decreasing SnRK1 activity concomitant with gradual increasing TOR activity following sucrose resupply. Three-day-old SA seedlings germinated on half-strength Murashige and Skoog (½ MS) medium containing 1% sucrose were transferred to ½ MS liquid medium without supplementation of sucrose and starved for 4 d in dark. Seedlings were then subsequently resupplied with 1% sucrose in the presence or absence of 1 μM TOR-specific inhibitor Torin2 and sampled at 0.5–4 h after sucrose resupply. Representative immunoblots show phosphorylated RPS6 (pRPS6), total RPS6, phosphorylated S6K1-HA (pS6K1-HA), total S6K1-HA, phosphorylated ACC1-HA (pACC1-HA), and total ACC1-HA. Ponceau S staining of Rubisco serves as a protein loading control. Band intensities were quantified using GelAnalyzer 23.1.1 and normalized to the protein loading control. TOR and SnRK1 activities were quantified below based on the pRPS6/RPS6 protein and pACC1-HA/ACC1-HA ratios, respectively. Different letters with the same color indicate statistically significant differences among time points for TOR or SnRK1 activity (Tukey’s multiple comparisons test, n=3, *P* < 0.05).

To simultaneously monitor both TOR and SnRK1 activities *in vivo*, we developed an Arabidopsis dual-reporter line by crossing an *S6K1-HA* transgenic line (Xiong and Sheen, 2012) with a transgenic SnRK1 reporter line expressing two tandem rat ACC1 peptides fused to GFP and HA tags (Sanagi et al., 2021; Avidan et al., 2023). Lines exhibiting stable expression for both S6K1-HA and ACC1 were selected and are hereafter referred to as the SA line (**Supplementary Figure 1**). Here, the ratio of phosphorylated RPS6 to total RPS6 (pRPS6/RPS6), a direct downstream readout of S6K1 (Chen et al., 2018), was used to quantify *in vivo* TOR activity. Phosphorylation-induced band shift of S6K1-HA (Van Leene et al., 2019; Persyn et al., 2024) or phosphorylation of S6K1-HA directly detected by anti-phospho-S6K1 antibody serves as an additional TOR activity readout. As shown in **Figure 1**, phosphorylation of RPS6 was closely correlated with phosphorylation of S6K1-HA (**Figure 1**). Moreover, overexpression of S6K1 markedly increased sensitivity of the pRPS6/RPS6 readout for TOR activity (**Supplementary Figure 2**), enabling reliable detection of dynamic changes in TOR activity under short-term experimental manipulations. Furthermore, no observable phenotypic differences were detected between *S6K1-HA* overexpression and wild-type plants under the experimental conditions used in this study, suggesting that *S6K1-HA* overexpression has minimal impact on plant growth and development. *In vivo* SnRK1 activity was quantified by measuring the ratio of phosphorylated ACC1-GFP-HA to total ACC1-GFP-HA (pACC1-HA/ACC-HA).

### 3.2 Negative relationship between TOR and SnRK1 activity during sucrose-mediated TOR activation

In plants, SnRK1 is activated under conditions of declining cellular energy and promotes metabolic reprogramming favoring catabolism over anabolism whereas TOR is activated by sugars such as glucose and sucrose (Xiong et al., 2013; Van Leene et al., 2019). We first examined the dynamics of SnRK1 and TOR activities during the transition from carbon starvation to carbon abundance conditions. Following sucrose resupply, SnRK1 activity in carbon-starved SA seedlings was rapidly suppressed i.e., within 30 min and remained low for up to 4 h. In contrast, TOR activity gradually increased over the same period (**Figure 1**), revealing an overall negative relationship between TOR and SnRK1 during the transition from carbon starvation to carbon abundance. However, the temporal patterns of their activities indicate that TOR and SnRK1 are not strictly inversely correlated throughout this process. Notably, supplementation of the TOR-specific inhibitor Torin2 during sucrose resupply abolished sucrose-mediated TOR activation but did not affect sucrose-induced suppression of SnRK1, indicating that sucrose-mediated SnRK1 suppression occurs mostly independent of TOR activity (**Figure 1**). Taken together, these results suggest the observed negative relationship between TOR and SnRK1 is more likely mediated indirectly by sucrose or cellular carbon status rather than by direct antagonistic interaction between the two kinase complexes.

### 3.3 Context-dependent negative relationship between TOR and SnRK1 activity under dark conditions

**Figure 2:**
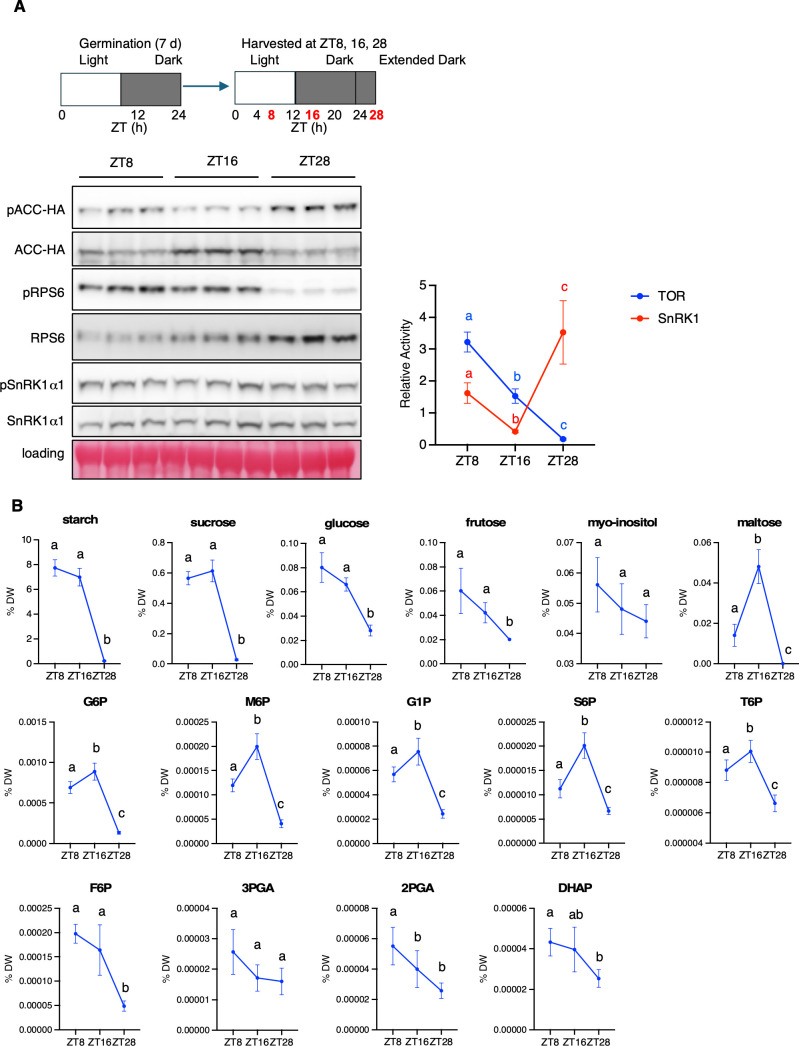
Partial negative relationship between TOR and SnRK1 activity under dark conditions. **(A)** Seven-day-old S6K1-HA/ACC1 (SA) seedlings grown under a 12/12 h day/night photoperiod were harvested at ZT8 (zeitgeber time, 8 h after the dawn), ZT16 (4 h into the dark), and ZT28 (16 h into the dark including 4 h of extended darkness). TOR activity decreased in the dark and was further reduced during extended darkness, whereas SnRK1 activity was initially suppressed in the dark but activated under the extended darkness. Representative immunoblots show pACC1-HA, ACC1-HA, pRPS6, RPS6, phosphorylated SnRK1α1 (pSnRK1α1) and total SnRK1α1.TOR and SnRK1 activities were quantified on the right based on the pRPS6/RPS6 and pACC1-HA/ACC1-HA ratios, respectively. Different letters with the same color indicate statistically significant differences among time points for TOR or SnRK1 activity (Tukey’s multiple comparisons test, n=3, *P* < 0.05). **(B)** Metabolite analysis showing changes in major carbon metabolites at ZT8, 16 and 28. Different letters in the same color indicate statistically significant differences in indicated metabolite among time points (Tukey’s multiple comparisons test, n=5, *P* < 0.05). Fructose-6-phosphate (F6P), 2-phosphoglyceric acid (2PGA), 3-phosphoglyceric acid (3PGA), dihydroxyacetone phosphate (DHAP), glucose-6-phosphate (G6P), maltose-6-phosphate (M6P), glucose-1-phosphate (G1P), sucrose-6-phosphate (S6P), trehalose-6-phosphate (T6P).

Extended darkness has previously been shown to activate SnRK1 (Pedrotti et al., 2018; Avidan et al., 2023). To further examine the relationship between TOR and SnRK1 activities with carbon availability, we monitored their activities at different time points across the diurnal cycle, including an extended dark period. SA plants grown under a 12h light/12h dark photoperiod were harvested at ZT8 (zeitgeber time, 8 h after dawn), ZT16 (4 h into the dark), and ZT28 (16 h into the dark including 4 h of extended darkness). TOR activity decreased at ZT16 and was further reduced at ZT28 (during the extended darkness), whereas SnRK1 activity was initially suppressed at ZT16 but became activated at ZT28 (**Figure 2A**), indicating a partial negative relationship between the two kinases under dark conditions.

To further interpret TOR and SnRK1 activities in the context of cellular carbon status under darkness, metabolomic analyses were performed to quantify the major carbohydrate metabolites at ZT8, ZT16 and ZT28. Two distinct metabolic trends were observed (**Figure 2B**). First, levels of carbohydrates, including starch, sucrose, glucose, fructose, fructose-6-phosphate (F6P), 2-phosphoglyceric acid (2PGA), and dihydroxyacetone phosphate (DHAP) progressively decreased during darkness, correlating with reduced TOR activity. Second, the contents of maltose and several phosphorylated carbohydrates including glucose-6-phosphate (G6P), maltose-6-phosphate (M6P), glucose-1-phosphate (G1P), sucrose-6-phosphate (S6P), and T6P increased at ZT16 but declined at ZT28, displaying an inverse correlation with SnRK1 activity. Taken together, these results suggest that TOR and SnRK1 are not governed by a simple antagonistic relationship and their functional interplay appears to be strongly influenced by cellular carbon status.

### 3.4 Context-dependent positive relationship between TOR and SnRK1 activity under the nitrogen starvation

**Figure 3:**
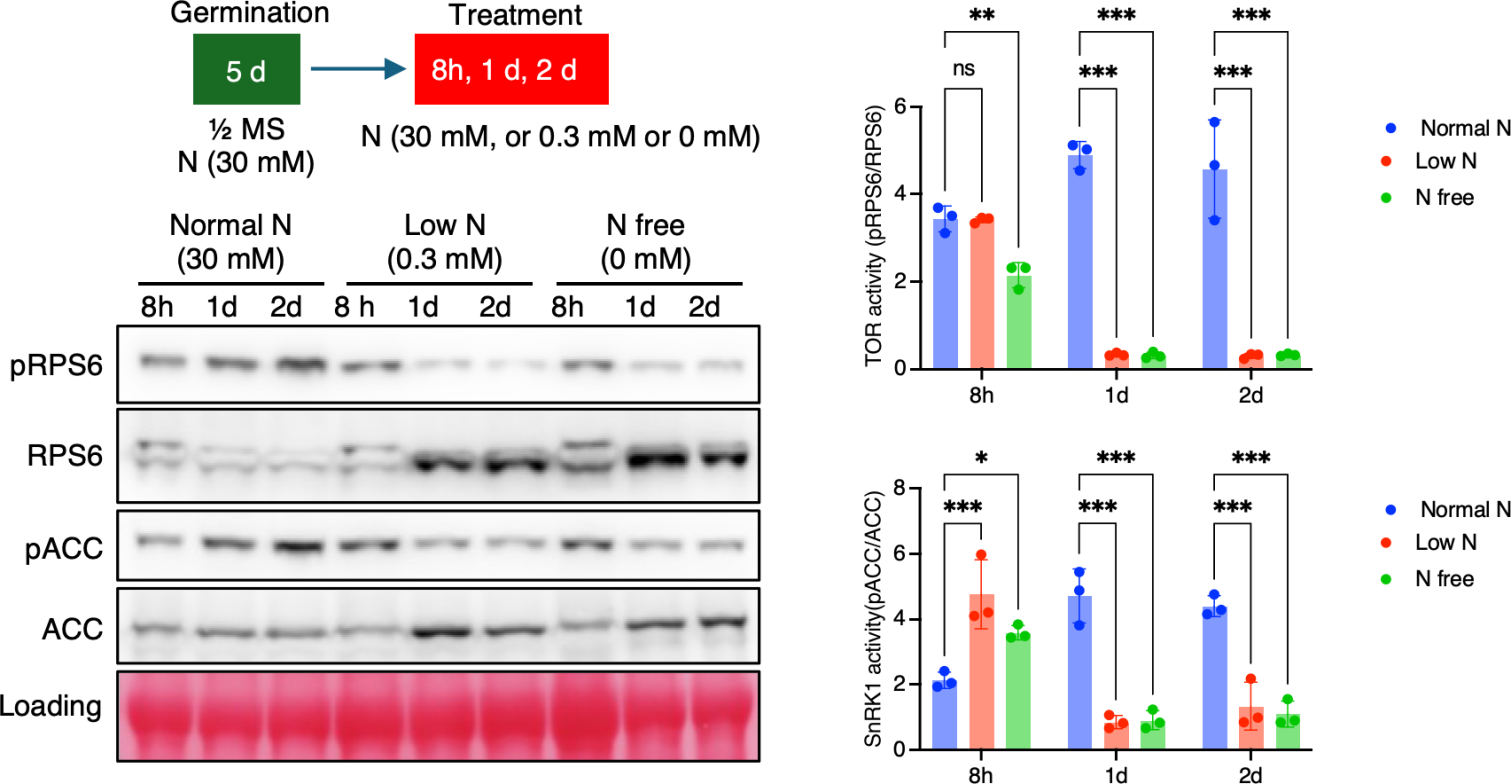
Partial positive relationship between TOR and SnRK1 activity under the nitrogen starvation. Five-day-old S6K1-HA/ACC1 seedlings germinated on ½ MS solid medium (1% sucrose) containing 30 mM of nitrogen were transferred to ½ MS liquid medium (1% sucrose) containing 30 mM or 0.3 mM or 0 mM nitrogen and harvested at the indicated time points. Representative immunoblots show pACC1-HA, ACC1-HA, pRPS6, and RPS6. TOR and SnRK1 activities were quantified as indicated. Statistical significance was determined by Tukey’s multiple comparisons test (n=3, *P < 0.033; **P < 0.002, ***P < 0.001, ns, not significant).

Low nitrogen (N) availability has previously been shown to reduce SnRK1 activity (Sanagi et al., 2021), and TOR activity (Liu et al., 2021; Persyn et al., 2024). Recently, Persyn et al. (2024) showed SnRK1 activity is activated in Arabidopsis cell culture overexpressing S6K1-HA during two days of nitrogen starvation, as evidenced by the transcriptional upregulation of SnRK1 target genes, including dark-inducible 1 (DIN1), trehalose-6-phosphate synthase 8 (TPS8), and TPS9 (Persyn et al., 2024). Using a SPARK sensor, they also revealed divergent SnRK1 activity patterns between shoot and root tissue of Arabidopsis seedlings, with increased activity in shoot and decreased activity in root after 3 d of nitrogen starvation. To address these discrepances, we examined the dynamics of both TOR and SnRK1 activities during N starvation in SA seedlings. Consistent with previous reports, TOR activity was gradually repressed over a 2-d period in plants grown under N-free conditions compared with normal N conditions (30 mM). The reduction in TOR activity was delayed in plants grown under low N conditions (0.3 mM). In contrast, SnRK1 activity increased following transfer to low N or N free conditions but became suppressed after 1 day of N starvation (**Figure 3**). Consistent with the repression of both TOR and SnRK1 activities, seedlings grown under N-deficient conditions for 1 d or 2 d exhibited growth arrest and stress-associated phenotypes, including anthocyanin accumulation (**Supplementary Figure 3**). Strong reduction of TOR and SnRK1 activity under N starvation implied that prolonged N deficiency exerts a dominant regulatory effect that overrides any antagonistic interplay between the two kinases under these conditions.

### 3.5 *In vitro* kinase assays demonstrate that SnRK1α1 directly suppresses TOR activity whereas immunoprecipitated TOR does not affect SnRK1α1 activity

**Figure 4:**
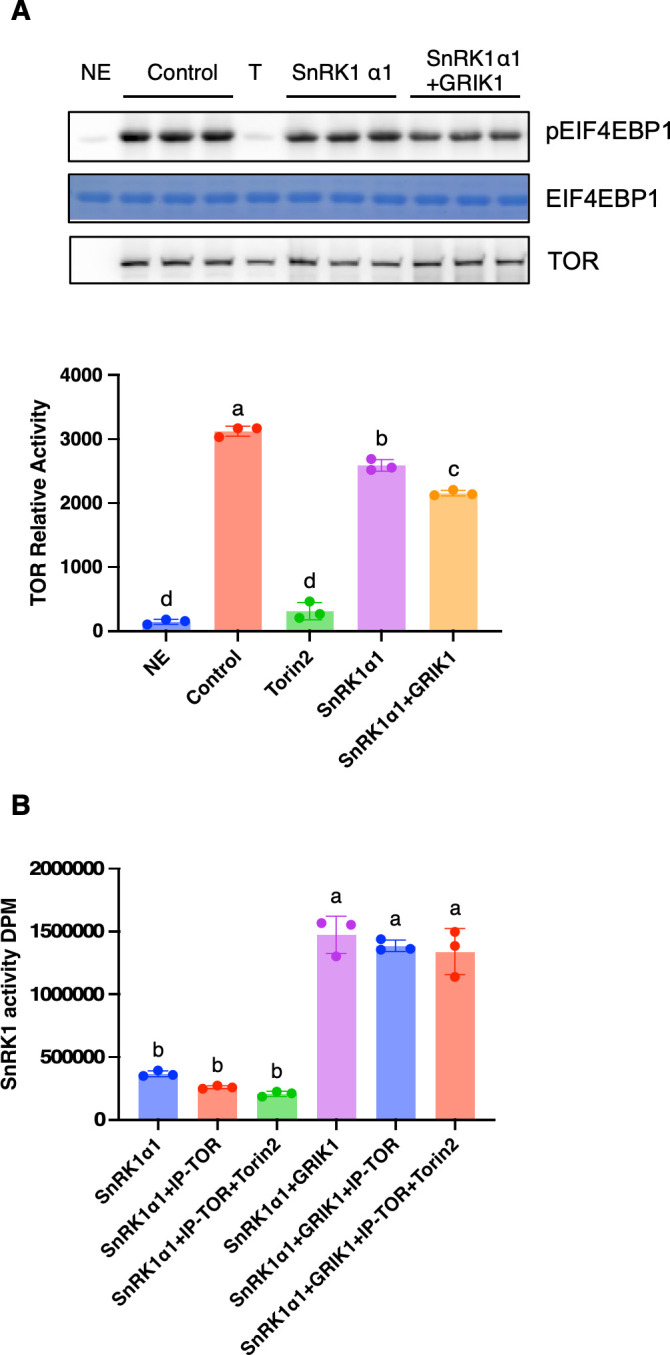
*In vitro* kinase assays demonstrated that SnRK1α1 directly suppresses TOR activity whereas IP-TOR does not affect SnRK1α1 activity. **(A)** In a 25-μL TOR kinase reaction, immunoprecipitated TOR (IP-TOR, 1 μg) from 7-d-old wild-type seedlings was incubated with TOR substrate EIF4EBP1 (1 μg) for 15 min at 30 °C, constituting the basal reaction (Control). Reactions lacking IP-TOR (NE, no enzyme) or containing Torin2 (T) served as negative controls. Recombinant SnRK1α1 (1 μg) or GRIK1-activated SnRK1α1 (1 μg SnRK1α1 plus 1 μg GRIK1) was added to the basal reaction to test the direct effect of SnRK1 on TOR activity. Phosphorylated EIF4EBP1 was detected with a phospho-specific antibody against EIF4EBP1 (pT37). Equal substrate loading was verified by Coomassie, equal TOR input was confirmed by immunoblotting with anti-TOR antibodies. TOR activity was quantification below. Different letters above bars indicate statistically significant differences (Tukey’s multiple comparisons test, n=3, *P* < 0.05). **(B)** *In vitro* SnRK1 kinase assays were performed by incubating recombinant SnRK1α1 (1 μg) or GRIK1-activated SnRK1α1 (1 μg SnRK1α1 plus 1 μg GRIK1) with SnRK1 target peptide derived from sucrose phosphate synthase (SPS) in the presence of [γ-^32^P] ATP for 5 min at 30 °C. IP-TOR (1 μg) was added to SnRK1 kinase reaction in the presence or absence of 1 μM of Torin2 to test the direct impact of TOR on SnRK1 activity. SnRK1 activity was determined by measuring incorporation of ^32^P from [γ-^32^P] ATP into SPS peptide and expressed as disintegrations per minute (DPM). Different letters above bars indicate statistically significant differences (Tukey’s multiple comparisons test, n=3, *P* < 0.05).

To examine the direct interplay between TOR and SnRK1, we performed *in vitro* protein kinase assays using immunoprecipitated TOR (IP-TOR) and recombinant SnRK1α1. For TOR kinase assays, TOR was immunoprecipitated from Arabidopsis seedlings using a TOR specific antibody (Liu et al., 2025a) and incubated with a recombinant TOR substrate EIF4EBP1 for 15 min at 30 °C. TOR activity was quantified based on the phosphorylation of EIF4EBP1, as detected using a phospho-specific antibody against EIF4EBP1 (pT37) (Xiong et al., 2013). IP-TOR showed robust phosphorylation of EIF4EBP1, which was abolished in reactions lacking IP-TOR or supplemented with Torin2, and enhanced by addition of recombinant constitutively active ROP2 (CA-ROP2), a known TOR activator (Schepetilnikov et al., 2017) (**Supplementary Figure 4**), while both SnRK1α1 and GRIK1-activated SnRK1α1 (SnRK1α1 plus its activating kinase GRIK1) cannot phosphorylate EIF4EBP1 (**Supplementary Figure 5**). These controls validated the specificity of the TOR kinase assay. Notably, supplementation with either recombinant SnRK1α1 or GRIK1-activated SnRK1α1 significantly reduced TOR activity by approximately 17% and 30%, respectively (**Figure 4A**), indicating SnRK1 can directly inhibit TOR activity *in vitro*.

To assess whether TOR directly regulates SnRK1 activity, we next performed *in vitro* SnRK1 kinase assays by incubating recombinant SnRK1α1 or GRIK1-activated SnRK1α1 with a substrate peptide derived from sucrose-phosphate synthase (SPS) (Zhai et al., 2018). SnRK1 activity was determined by measuring incorporation of ^32^P from [γ-^32^P] ATP into the SPS peptide. As expected, GRIK1-activated SnRK1α1 displayed approximately threefold higher activity relative to SnRK1α1 alone. In contrast, supplementation of IP-TOR had no significant effect on activity of either SnRK1α1 alone or GRIK1-activated SnRK1α1, indicating that TOR does not directly regulate SnRK1 activity (**Figure 4B**), consistent with the observation in **Figure 1** that sucrose-mediated SnRK1 suppression was independent of TOR activity.

## 4 Discussion

TOR and SnRK1 have been widely regarded as central regulators of plant growth and metabolism that function antagonistically in response to nutrient and energy availability. This “yin–yang” model, supported by genetic, transcriptomic, and biochemical evidence has shaped the current understanding of plant energy signaling (Baena-González and Hanson, 2017; Rodriguez et al., 2019). However, our results reveal a more nuanced and context-dependent relationship between TOR and SnRK1 activities.

### 4.1 TOR and SnRK1 exhibit conditional, but not universal, negative relationships *in vivo*

Using an Arabidopsis dual-reporter line that enables simultaneous monitoring of TOR and SnRK1 activities *in vivo*, we show that negative relationships between the two kinases emerge primarily under specific nutritional contexts. During the transition from carbon starvation to carbon abundance, sucrose resupply led to rapid suppression of SnRK1 activity and progressive activation of TOR. Although this pattern is consistent with an antagonistic relationship, the temporal dynamics indicate that TOR and SnRK1 activities are not strictly inversely correlated. Importantly, inhibition of TOR by Torin2 abolished sucrose-mediated TOR activation without affecting SnRK1 suppression, showing that sucrose-induced SnRK1 inactivation can be independent on TOR activity. These observations suggest that changes in cellular carbon status, rather than direct kinase antagonism, primarily drive the observed negative relationship during sucrose resupply.

A similarly conditional relationship was observed under dark conditions. Extended darkness led to strong repression of TOR and activation of SnRK1, consistent with declining carbon availability. However, during the early dark period, both TOR and SnRK1 activities were suppressed, which again is inconsistent with a simple antagonistic model. Metabolomic analyses revealed distinct carbohydrate signatures associated with TOR and SnRK1 activities, suggesting that each kinase responds to different aspects of cellular carbon metabolism. TOR activity correlated with depletion of major carbon reserves and glycolytic intermediates, whereas SnRK1 activity showed an inverse relationship with the abundance of phosphorylated sugars such as G6P, M6P, G1P, S6P, and T6P. Some of phosphorylated sugars, such as G6P, G1P and T6P are known inhibitors of SnRK1 (Zhang et al., 2009; Nunes et al., 2013; Blanford et al., 2024). Consistent with this, a recent study reported a stronger and widespread negative correlation between G6P levels and SnRK1 activity (Avidan et al., 2023).

Conditional relationship was also observed during nitrogen starvation. During the early phase of N deprivation, activation of SnRK1 coincided with repression of TOR. However, after one or two days of N depletion, activities of both kinases were markedly reduced, accompanied by growth arrest and anthocyanin accumulation in seedlings. These findings indicate that prolonged nitrogen deficiency suppresses both growth-promoting and energy-sensing pathways, overriding any antagonistic interaction between TOR and SnRK1.

The distinct dynamics pattern of TOR and SnRK1 activities in response to N versus C starvation likely reflect nutrient-specific modes of regulation, emphasizing the importance of nutrient context in shaping TOR–SnRK1 signaling relationships.

Regarding the discrepancies with the observations of Persyn et al. (2024), in which SnRK1 remained activated in Arabidopsis cell culture throughout 48 h N starvation, differences in experimental readouts may provide an explanation. In this study, SnRK1 activity was measured using a direct ACC-based reporter, whereas Persyn et al. relied on the expression of SnRK1 target genes. Gene expression responses may be highly sensitive to the activation of SnRK1 but less responsive to its repression and may also exhibit delayed dynamics.

### 4.2 SnRK1 influences TOR activity through both direct and indirect mechanisms

*In vitro* kinase assays further help clarify the directionality of TOR–SnRK1 interactions. Recombinant SnRK1α1 directly inhibited TOR kinase activity, whereas immunoprecipitated TOR had no detectable effect on SnRK1α1 activity, establishing a unidirectional, direct inhibitory capacity of SnRK1 with respect to TOR. However, the relatively modest inhibition observed *in vitro*, together with the context-dependent relationships observed *in vivo*, suggests that additional regulatory layers such as metabolic intermediates, hormonal signals, or subcellular compartmentalization can play dominant roles in shaping TOR signaling. Consistent with this view, abscisic acid (ABA) has been shown to modulate SnRK1 subcellular localization via SnRK2-dependent mechanisms, thereby influencing TOR signaling (Belda-Palazon et al., 2020; Belda-Palazón et al., 2022).

### 4.3 A model for TOR–SnRK1 coordination

**Figure 5:**
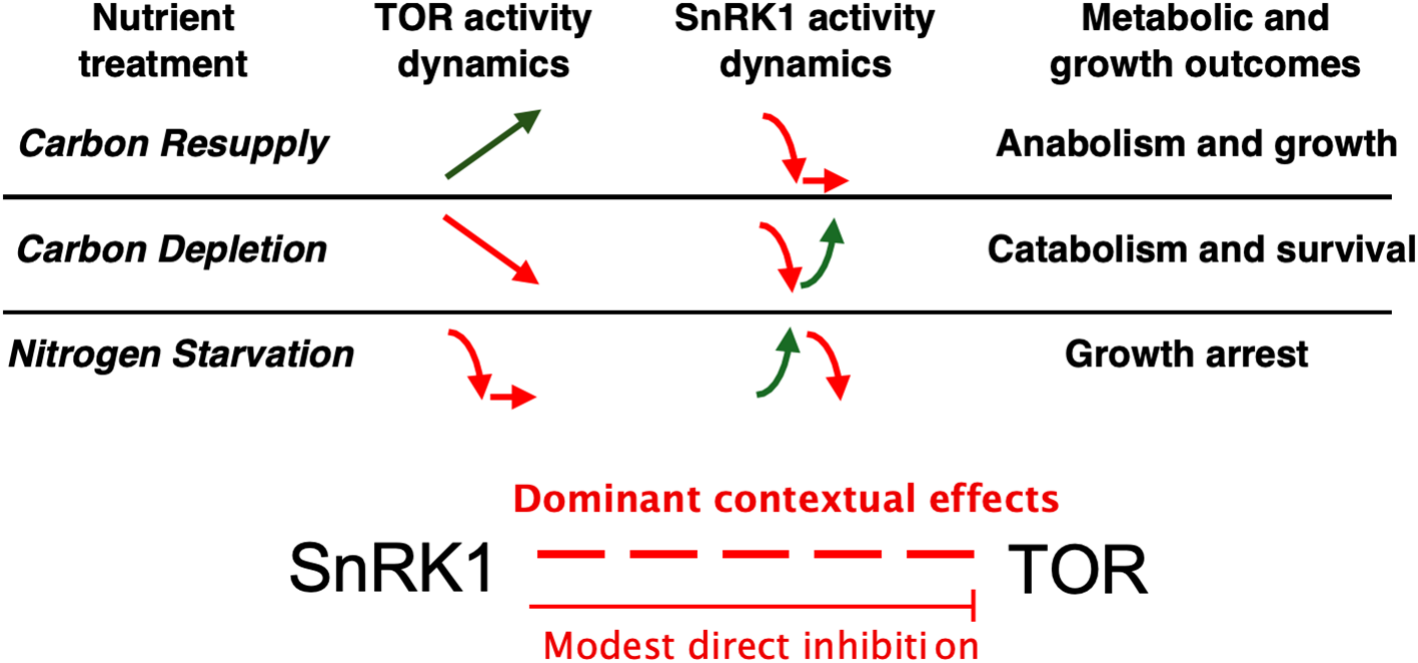
A summary schematic of nutrient treatments on the *in vivo* activity dynamics for TOR and SnRK1 and associated metabolic and growth outcomes.During the transition from carbon starvation to abundance, TOR activity gradually increases, in contrast, SnRK1 activity is rapidly repressed, leading to anabolism and growth.Under carbon depletion in the dark, TOR activity is gradually repressed, while SnRK1 activity is initially repressed and subsequently activated during extended darkness, leading to catabolism and a survival mode.During nitrogen starvation, TOR activity is repressed after one day, while SnRK1 is initially activated and subsequently repressed, leading to growth arrest.Green arrows indicate increased activity, red arrows, decreased activity.Direct inhibitory effect on TOR by SnRK1 is modest *in vitro*; indirect/contextual effects dominate *in vivo*.

Taken together, our findings support a model in which TOR and SnRK1 are coordinated primarily by cellular nutrient and metabolic context rather than functioning via a simple antagonistic mechanism (**Figure 5**). Although SnRK1 can directly inhibit TOR, it is insufficient to explain the diverse *in vivo* behaviors of the two kinases. Instead, TOR and SnRK1 respond to overlapping but distinct metabolic cues, leading to context-dependent coupling, uncoupling, or apparent antagonism. This flexible regulatory architecture likely evolved to enable plants to fine-tune growth and metabolism across rapidly changing environmental conditions.

In this study, SnRK1 activity was monitored using direct phosphorylation of the ACC reporter, whereas TOR activity was inferred through phosphorylation of the established downstream TOR target RPS6 in **Figure 2** and **Figure 3**. Therefore, the measurements should be interpreted as pathway-associated signaling dynamics rather than directly comparable catalytic outputs of the two kinases.

Although we did not observe significant growth differences between WT and S6K1-HA OX plants in our experimental conditions and pRPS6/RPS6 ratios were comparable in WT and S6K1-HA OX under the starved conditions, it is still possible that overexpression of S6K1 could distort native TOR signaling.

Because whole-seedling assays were used in this study, the measured activities likely represent averages across tissues with distinct responses. Future studies incorporating tissue- or cell type–specific reporters and combinations of different reporters will allow higher-resolution mapping of TOR–SnRK1 relationships.

## Data Availability

The raw data supporting the conclusions of this article will be made available by the authors, without undue reservation.

## Supplementary material

Supplementary Figure 1 Generation and validation of an Arabidopsis dual-reporter line for TOR and SnRK1 activity. An Arabidopsis dual-reporter line was generated by crossing an *S6K1-HA* transgenic line with a SnRK1-reporter line expressing two tandem rat ACC1 peptides fused to GFP and HA tags. Lines exhibiting stable expression of both S6K1-HA and ACC1-HA were selected for further analysis. Total protein was extracted from leaves of 4-week-old soil-grown plants. M, protein molecular weight makers. Each lane represents protein extracted from an individual plant. Both S6K1-HA and ACC1-HA were detected by probing with an anti-HA antibody.

Supplementary Figure 2 Enhanced sensitivity of the RPS6/RPS6 TOR activity readout in *S6K1-HA* overexpression line during sucrose-mediated TOR activation. Three-day-old WT and S6K1-HA seedlings germinated on ½ MS (1% sucrose) under long-day conditions were transferred to ½ MS liquid medium and starved for 4 d in the dark. Seedlings were subsequently resupplied with 1% sucrose and sampled at 0.5–4 h following sucrose resupply. Representative immunoblots show phosphorylated RPS6 (pRPS6) and total RPS6. TOR activity was quantified on the right based on pRPS6/RPS6 ratio. Different letters indicate statistically significant differences in TOR activity among time points for WT or *S6K1-HA* transgenic plants (Tukey’s multiple comparisons test, n=3, *P* < 0.05).

Supplementary Figure 3 Phenotypes of Arabidopsis S6K1-HA/ACC1 seedlings under nitrogen limitation. Phenotypes of 5-d-old Arabidopsis S6K1-HA/ACC1 seedlings grown in the half-strength MS liquid medium containing 30 mM, 0.3 mM or 0 mM of nitrogen for 1 d or 2 d.

Supplementary Figure 4 *In vitro* TOR kinase assay with immunoprecipitated TOR and recombinant TOR substrate protein EIF4EBP1. In a 25-μL TOR kinase reaction, immunoprecipitated TOR (IP-TOR, 1 μg) from 7-d-old wild-type seedlings was incubated with recombinant TOR substrate EIF4EBP1 (1 μg) for 15 min at 30 °C. Reactions lacking IP-TOR or containing Torin2 served as negative controls, whereas reactions supplemented with constitutively active ROP2 (CA-ROP2) served as a positive control. Phosphorylated EIF4EBP1 was detected with a phospho-specific antibody against EIF4EBP1 (pT37). Equal substrate loading was verified by Coomassie, equal TOR input was confirmed by immunoblotting with anti-TOR antibodies.

Supplementary Figure 5 *In vitro* kinase assay with recombinant SnRK1α1 and TOR substrate protein EIF4EBP1. In a 25-μL kinase reaction, recombinant SnRK1α1 or SnRK1α1 and GRIK1 was incubated with recombinant TOR substrate EIF4EBP1 (1 μg) for 15 min at 30 °C. Phosphorylated EIF4EBP1 was detected with a phospho-specific antibody against EIF4EBP1 (pT37). Equal kinases and substrate loading was verified by Coomassie as indicated (bottom).
