# Supplementary figures and images for: Context-dependent coordination of TOR and SnRK1 signaling under carbon and nitrogen perturbations
Source: Front Plant Sci. 2026 Jun 11;17:1862048. doi: 10.3389/fpls.2026.1862048 (PMC13294088; doi:10.3389/fpls.2026.1862048)

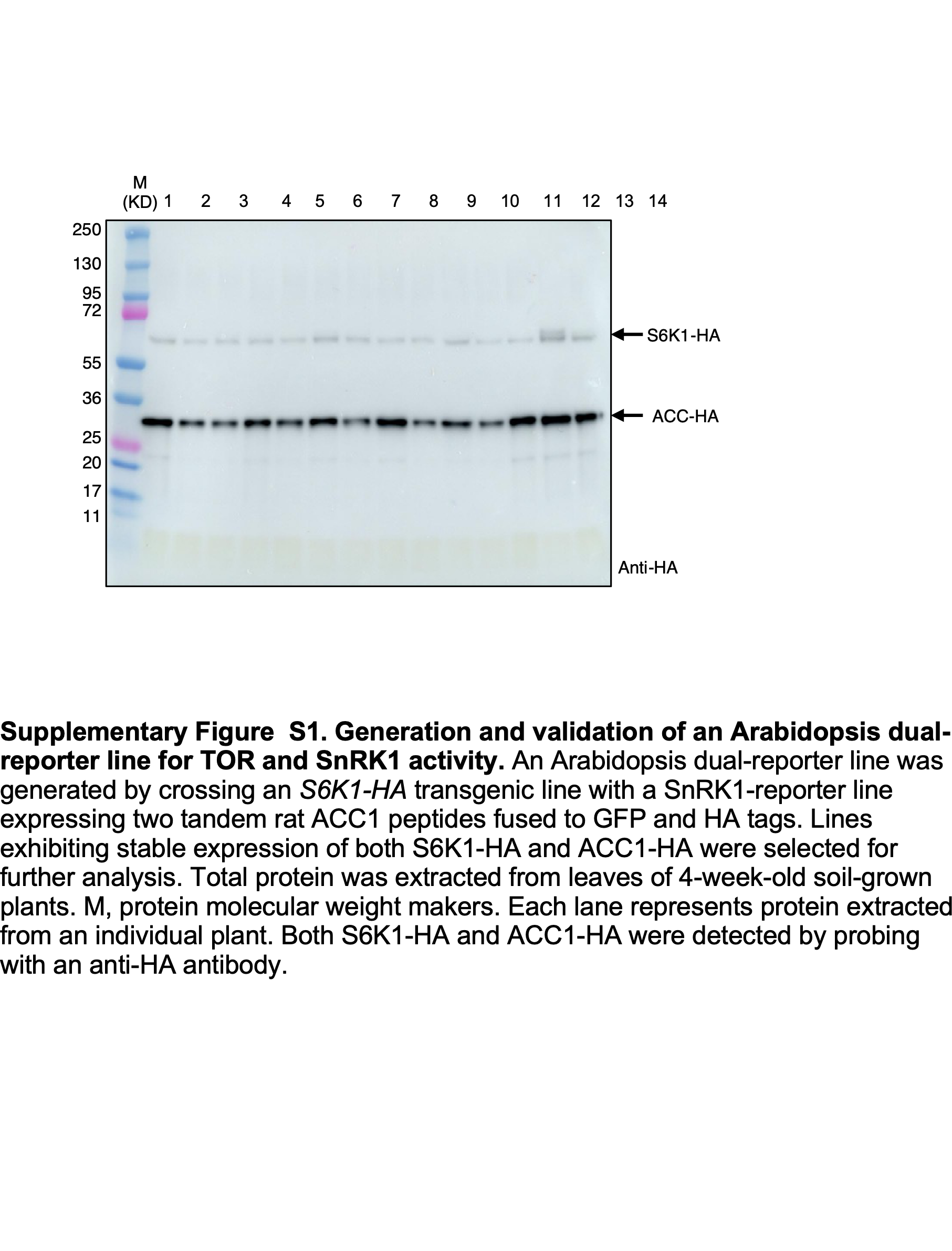

Supplement: Supplementary Figure 1 — Generation and validation of an Arabidopsis dual-reporter line for TOR and SnRK1 activity. An Arabidopsis dual-reporter line was generated by crossing an S6K1-HA transgenic line with a SnRK1-reporter line expressing two tandem rat ACC1 peptides fused to GFP and HA tags. Lines exhibiting stable expression of both S6K1-HA and ACC1-HA were selected for further analysis. Total protein was extracted from leaves of 4-week-old soil-grown plants. M, protein molecular weight makers. Each lane represents protein extracted from an individual plant. Both S6K1-HA and ACC1-HA were detected by probing with an anti-HA antibody. [file Image1.tif]

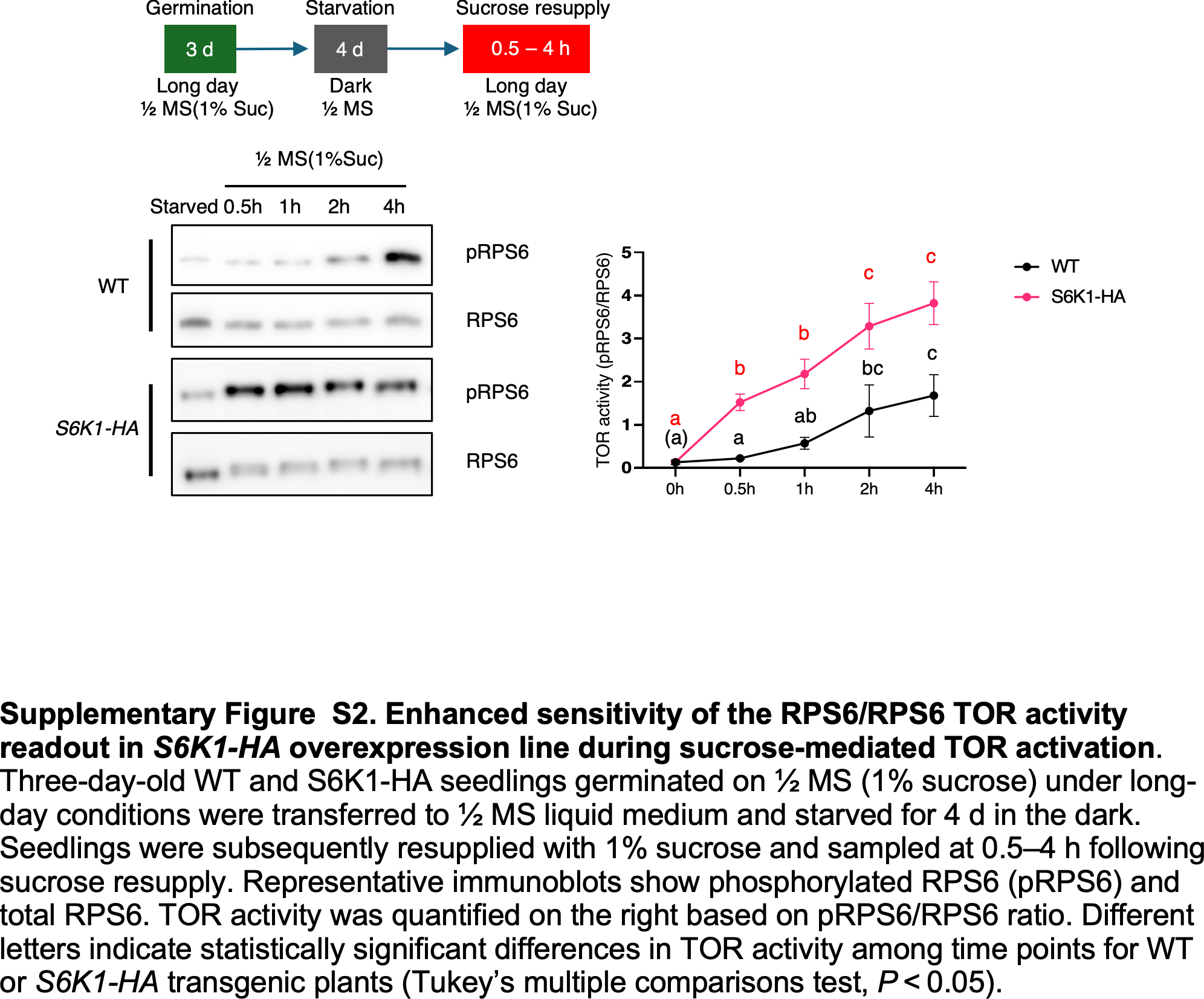

Supplement: Supplementary Figure 2 — Enhanced sensitivity of the RPS6/RPS6 TOR activity readout in S6K1-HA overexpression line during sucrose-mediated TOR activation. Three-day-old WT and S6K1-HA seedlings germinated on ½ MS (1% sucrose) under long-day conditions were transferred to ½ MS liquid medium and starved for 4 d in the dark. Seedlings were subsequently resupplied with 1% sucrose and sampled at 0.5–4 h following sucrose resupply. Representative immunoblots show phosphorylated RPS6 (pRPS6) and total RPS6. TOR activity was quantified on the right based on pRPS6/RPS6 ratio. Different letters indicate statistically significant differences in TOR activity among time points for WT or S6K1-HA transgenic plants (Tukey’s multiple comparisons test, n=3, P < 0.05). [file Image2.tif]

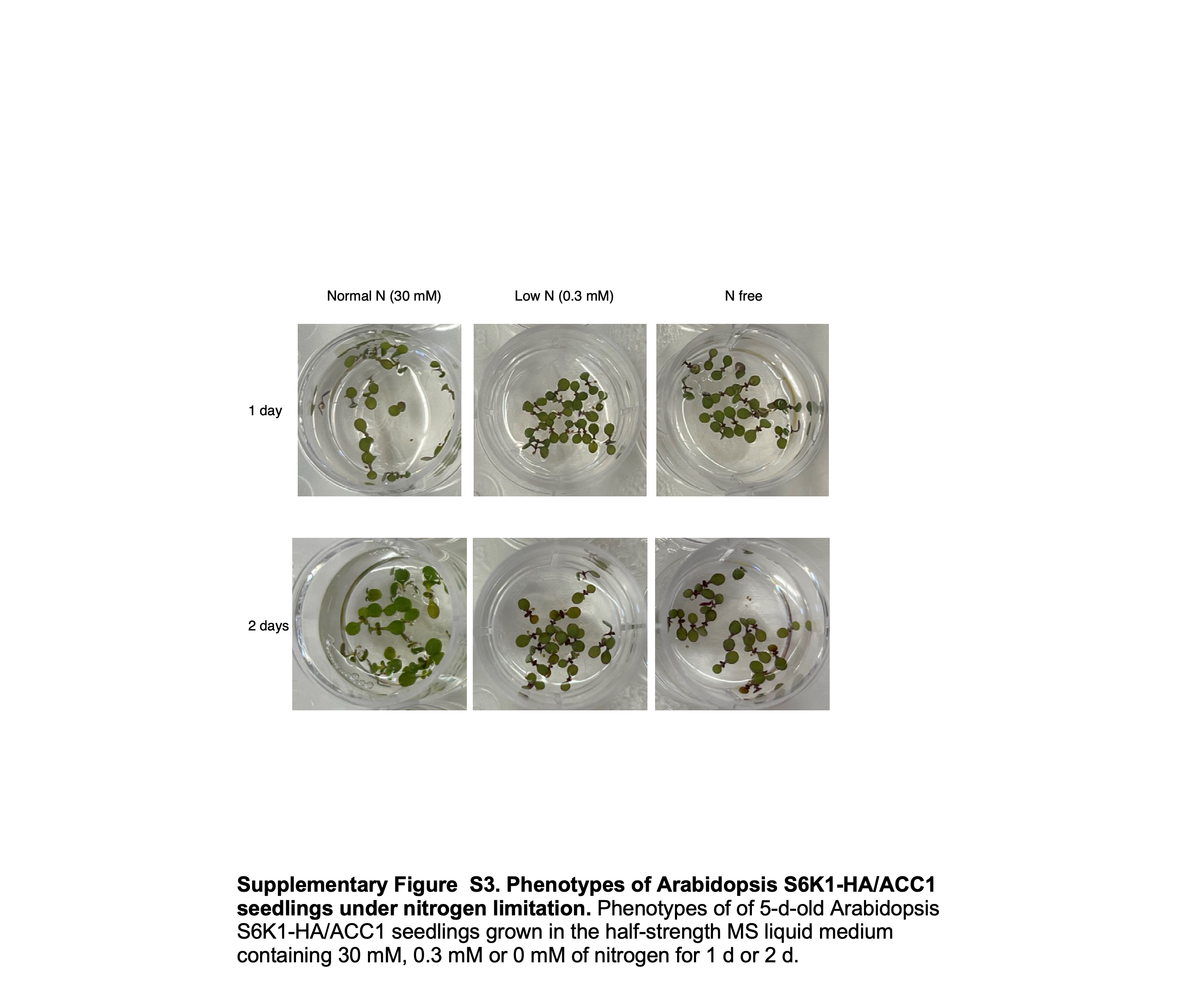

Supplement: Supplementary Figure 3 — Phenotypes of Arabidopsis S6K1-HA/ACC1 seedlings under nitrogen limitation. Phenotypes of 5-d-old Arabidopsis S6K1-HA/ACC1 seedlings grown in the half-strength MS liquid medium containing 30 mM, 0.3 mM or 0 mM of nitrogen for 1 d or 2 d. [file Image3.tif]

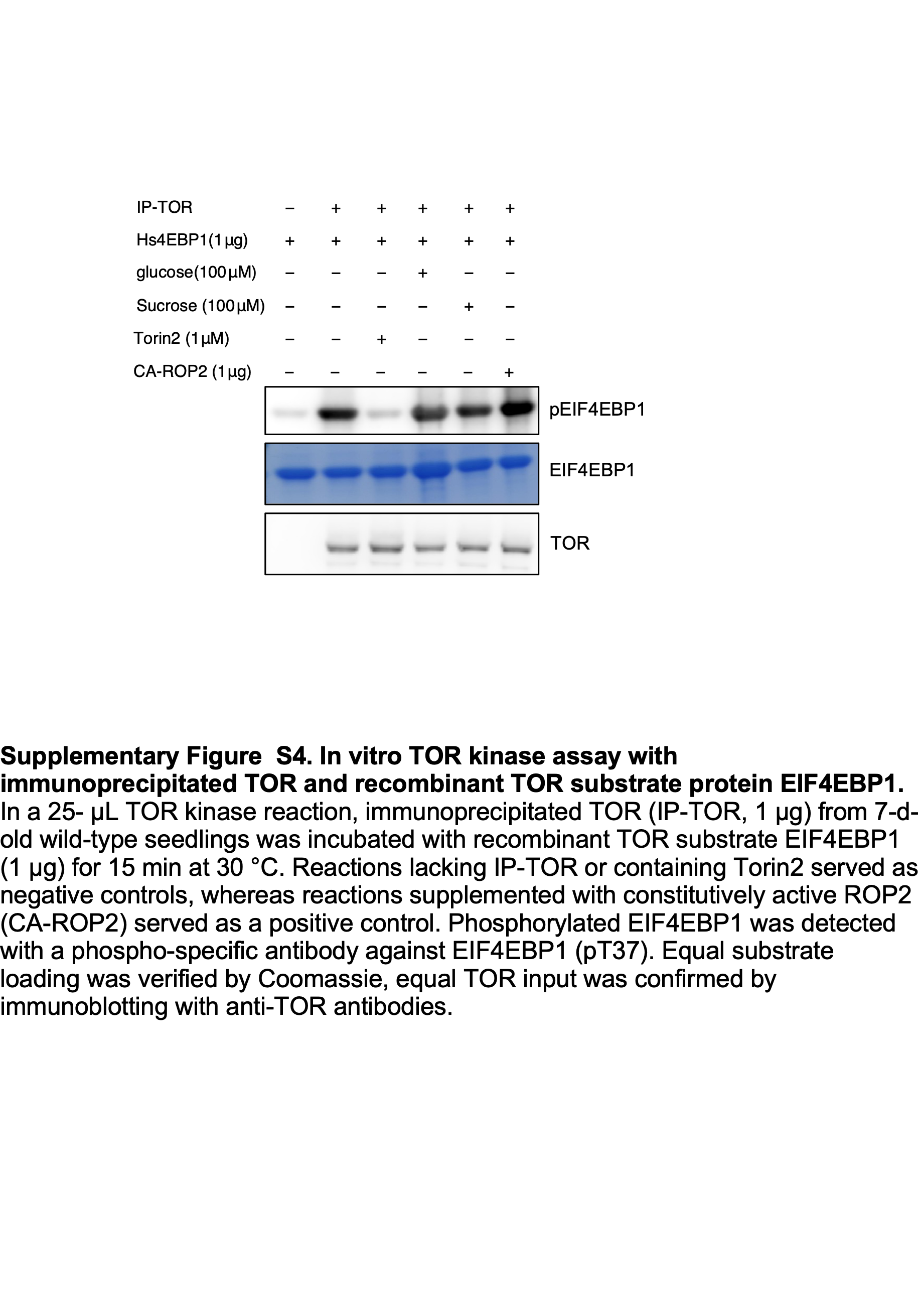

Supplement: Supplementary Figure 4 — In vitro TOR kinase assay with immunoprecipitated TOR and recombinant TOR substrate protein EIF4EBP1. In a 25-μL TOR kinase reaction, immunoprecipitated TOR (IP-TOR, 1 μg) from 7-d-old wild-type seedlings was incubated with recombinant TOR substrate EIF4EBP1 (1 μg) for 15 min at 30 °C. Reactions lacking IP-TOR or containing Torin2 served as negative controls, whereas reactions supplemented with constitutively active ROP2 (CA-ROP2) served as a positive control. Phosphorylated EIF4EBP1 was detected with a phospho-specific antibody against EIF4EBP1 (pT37). Equal substrate loading was verified by Coomassie, equal TOR input was confirmed by immunoblotting with anti-TOR antibodies. [file Image4.tif]

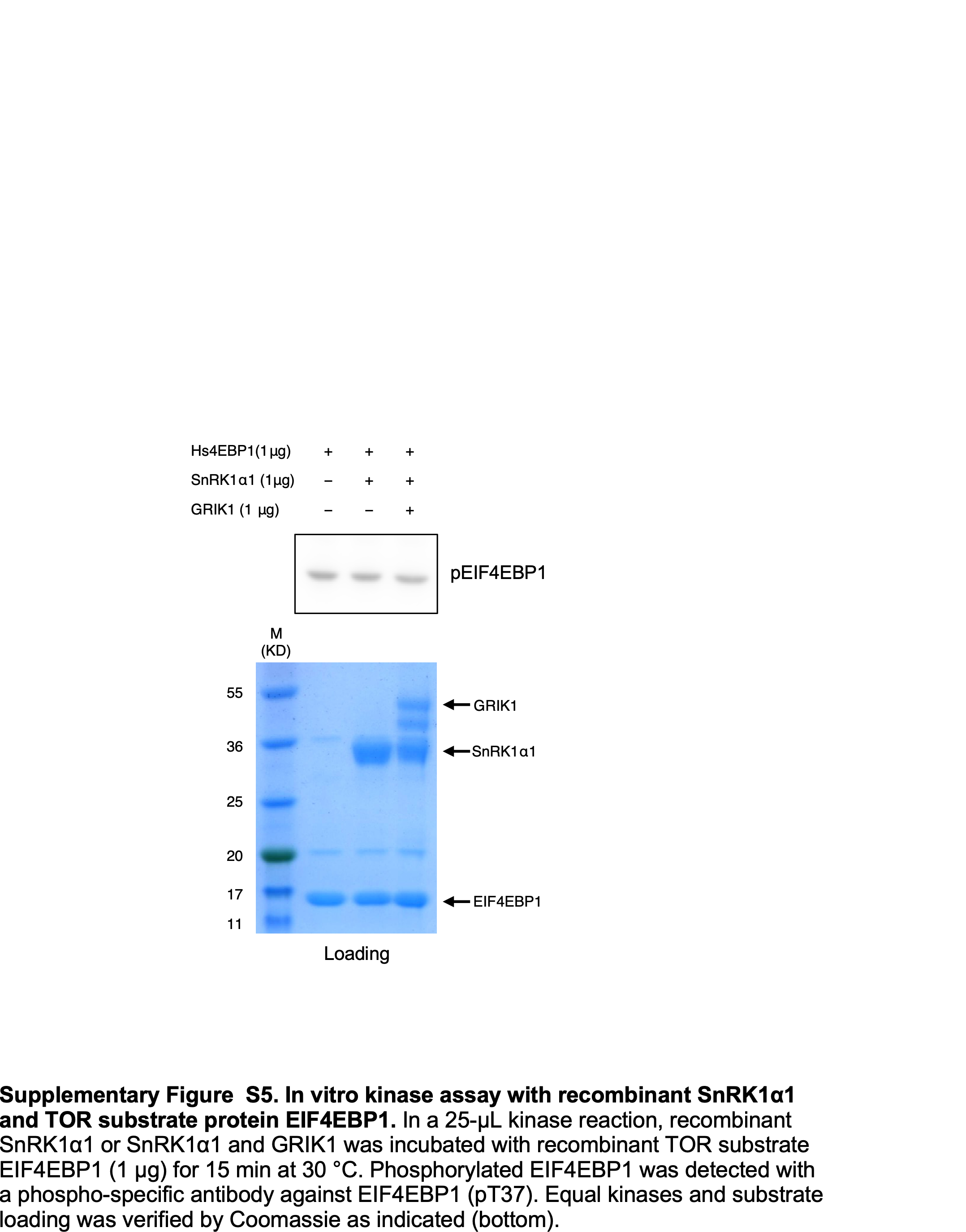

Supplement: Supplementary Figure 5 — In vitro kinase assay with recombinant SnRK1α1 and TOR substrate protein EIF4EBP1. In a 25-μL kinase reaction, recombinant SnRK1α1 or SnRK1α1 and GRIK1 was incubated with recombinant TOR substrate EIF4EBP1 (1 μg) for 15 min at 30 °C. Phosphorylated EIF4EBP1 was detected with a phospho-specific antibody against EIF4EBP1 (pT37). Equal kinases and substrate loading was verified by Coomassie as indicated (bottom). [file Image5.tif]
